# Supplementary material for: ABL Genomic Editing Sufficiently Abolishes Oncogenesis of Human Chronic Myeloid Leukemia Cells In Vitro and In Vivo
Source: Cancers (Basel). 2020 May 29;12(6):1399. doi: 10.3390/cancers12061399 (PMC7352505; doi:10.3390/cancers12061399)

Supplementary Information File

Raw figure of Figure 1I-BCR-ABL

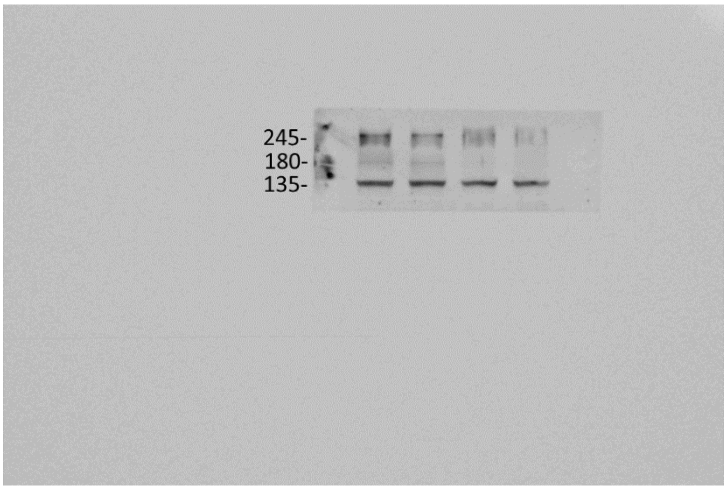

Raw figure of Figure 1I-c-PARP

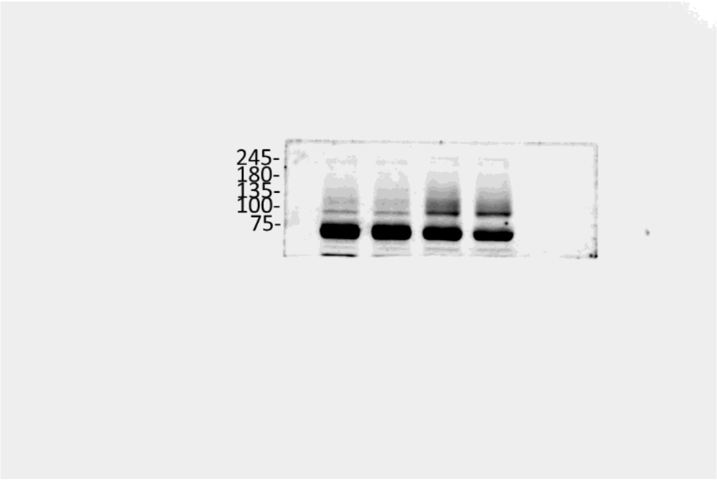

Raw figure of Figure 1I-P21

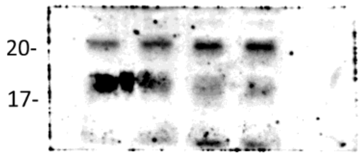

Raw figure of Figure 1I-GAPDH

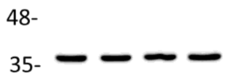

Raw figure of Figure 3A

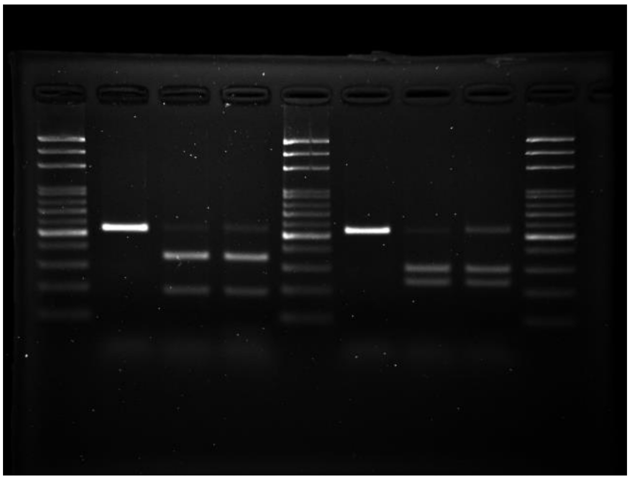

Raw figure of Figure 3B –BCR-ABL

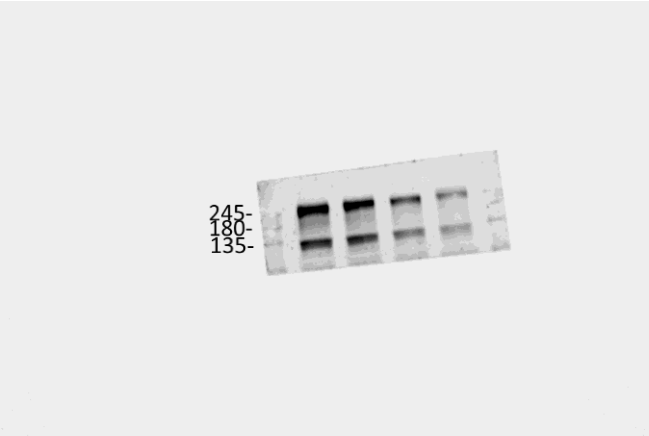

Raw figure of Figure 3B –c-PARP

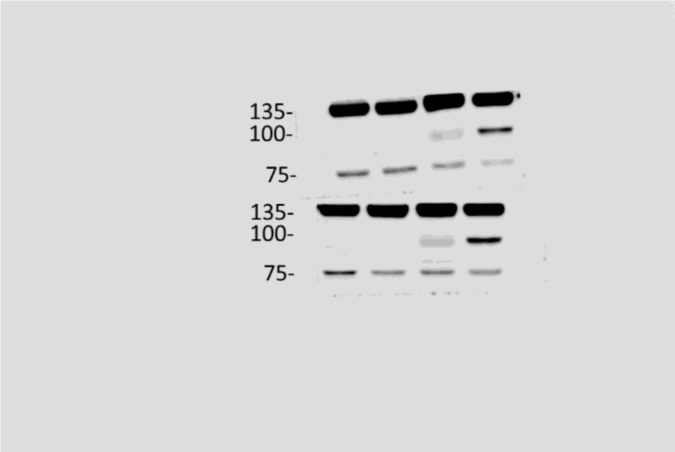

Raw figure of Figure 3B –P21

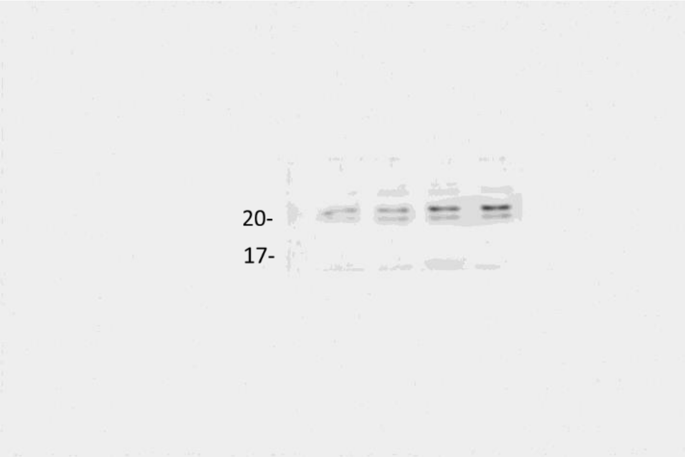

Raw figure of Figure 3B –GAPDH

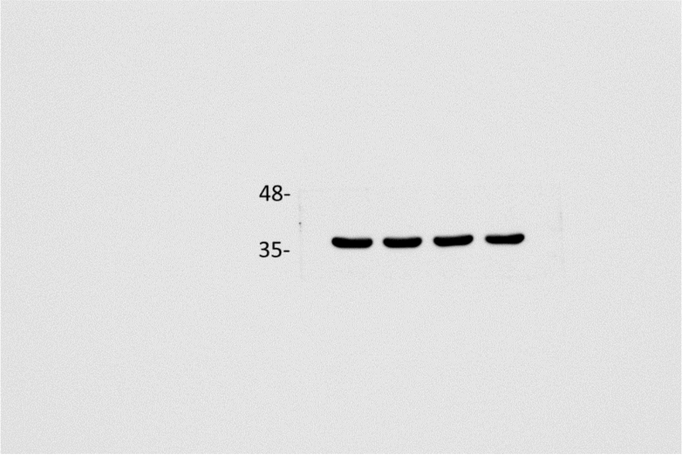

Raw figure of Figure 4A- mouse 2: SC_sgRNA; mouse 5: ABL sgRNA_2

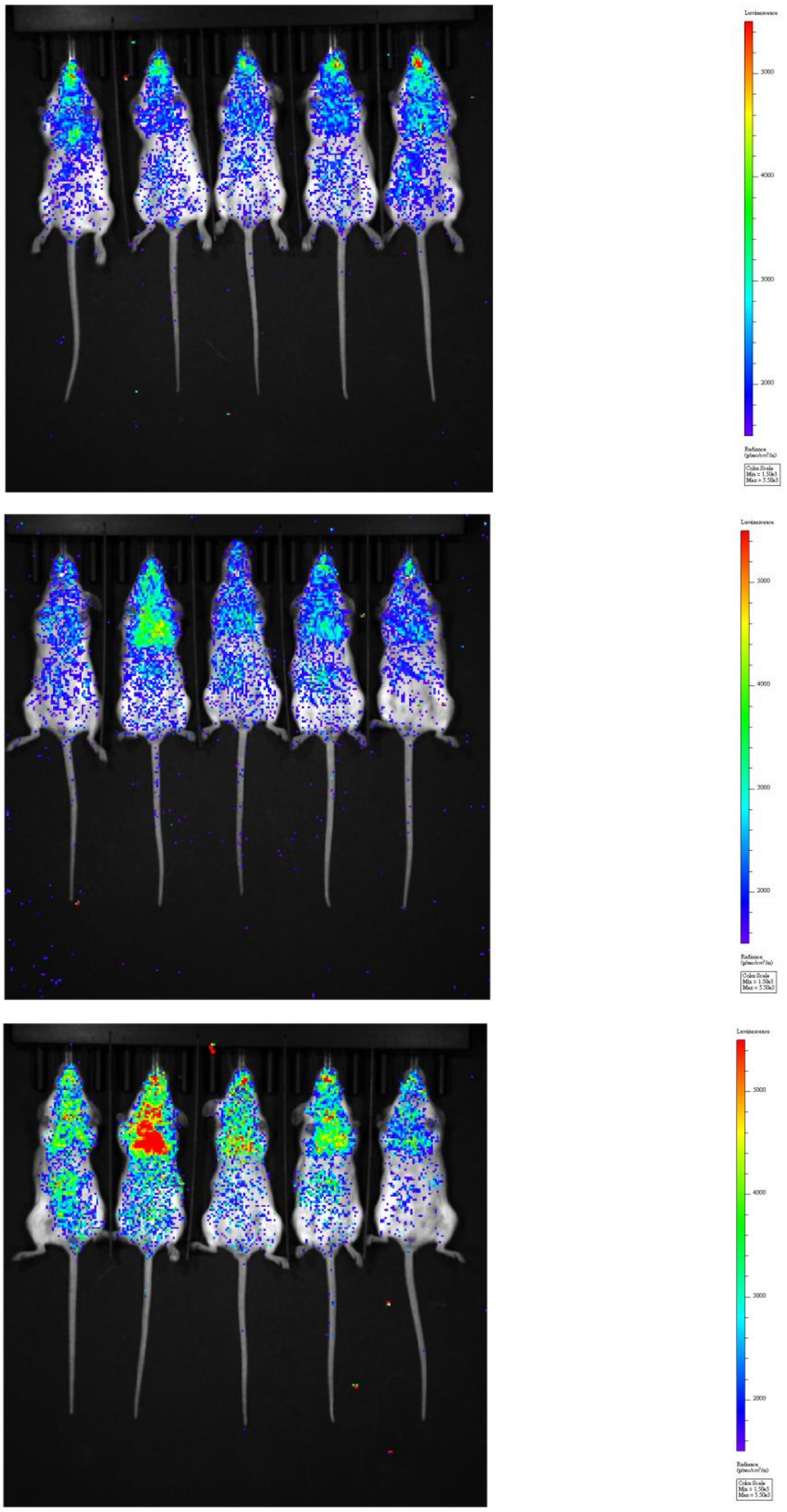

Raw figure of Figure 4B- mouse 2: SC_sgRNA; mouse 5: ABL sgRNA_2

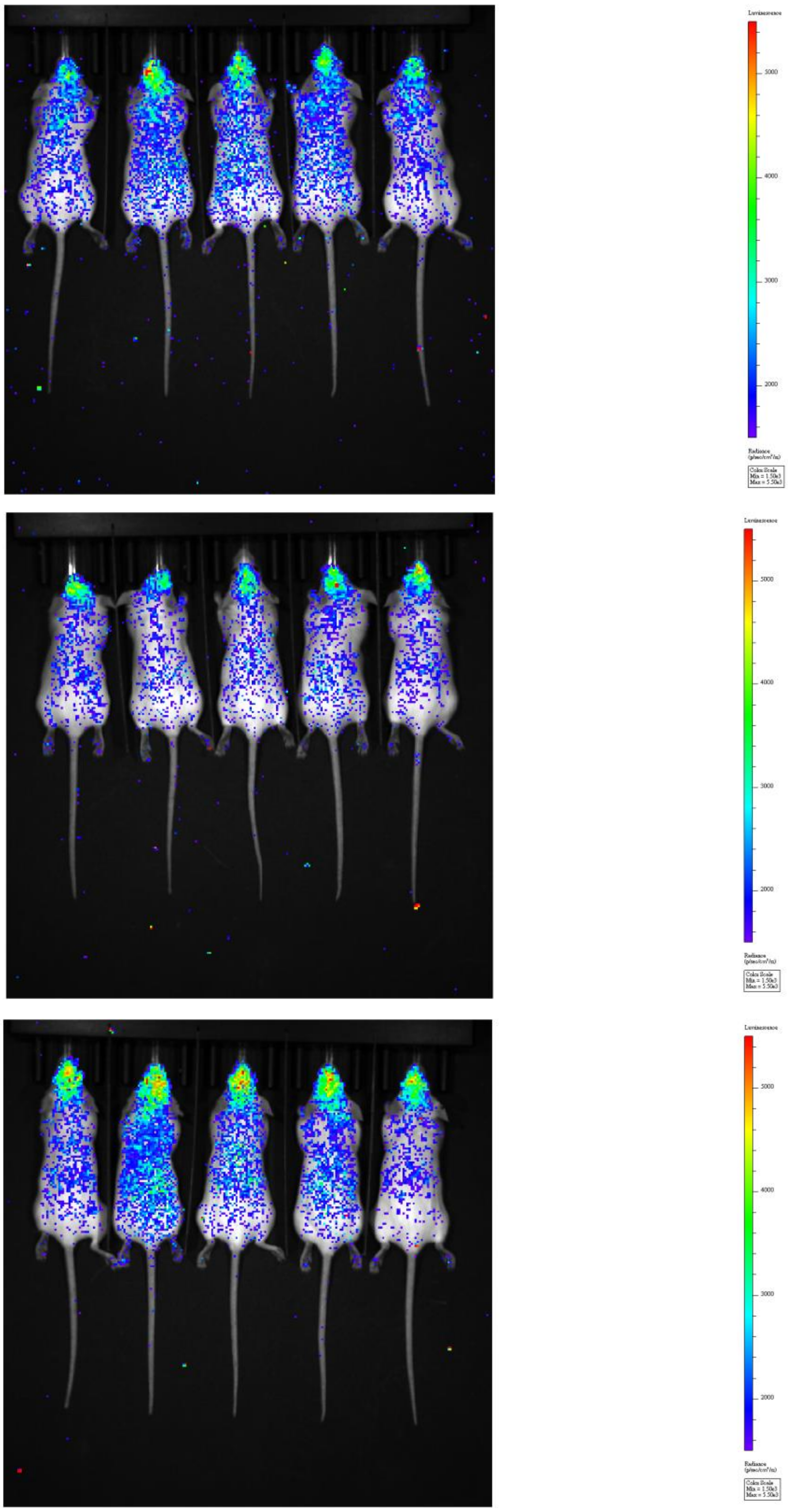

Raw figure of Figure 4E

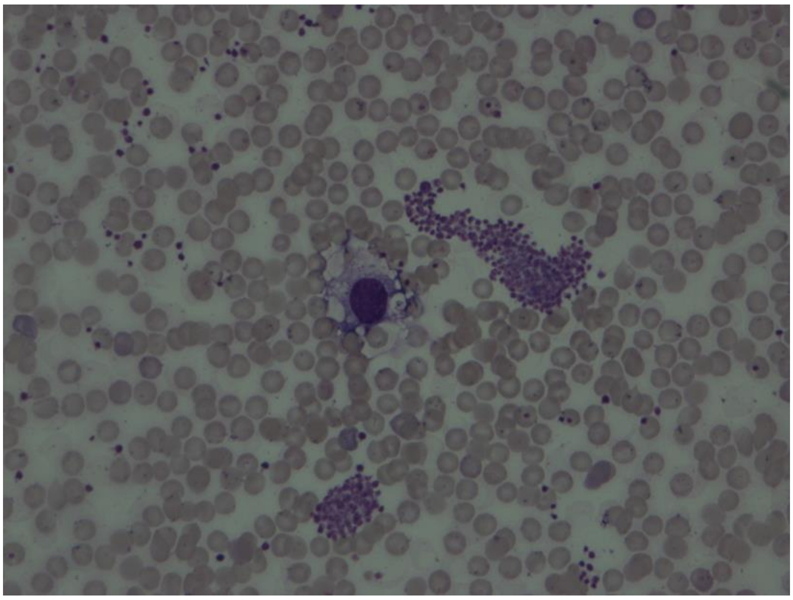

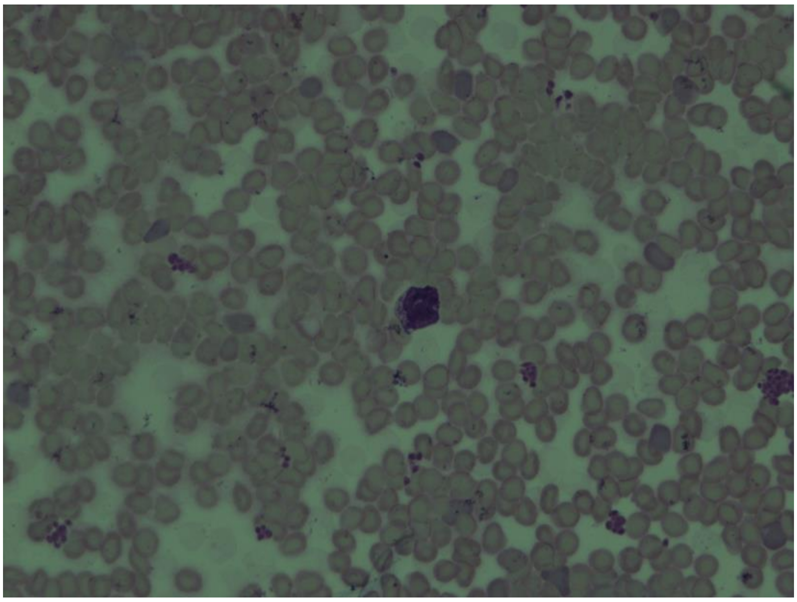

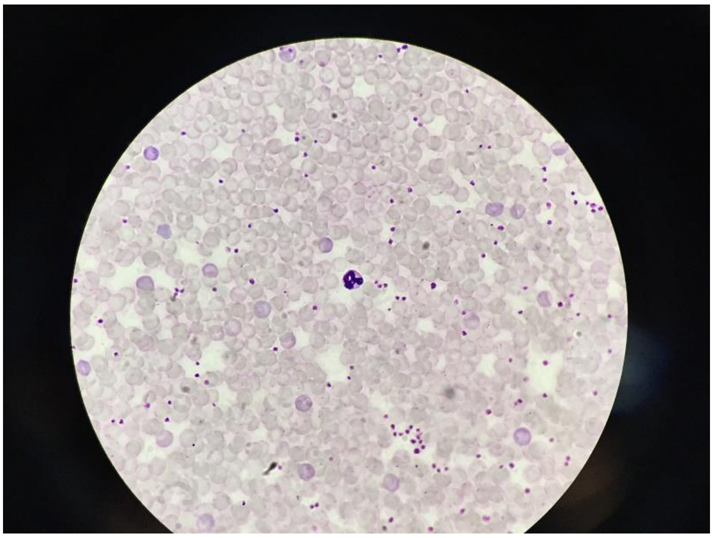

Raw figure of Figure 5C

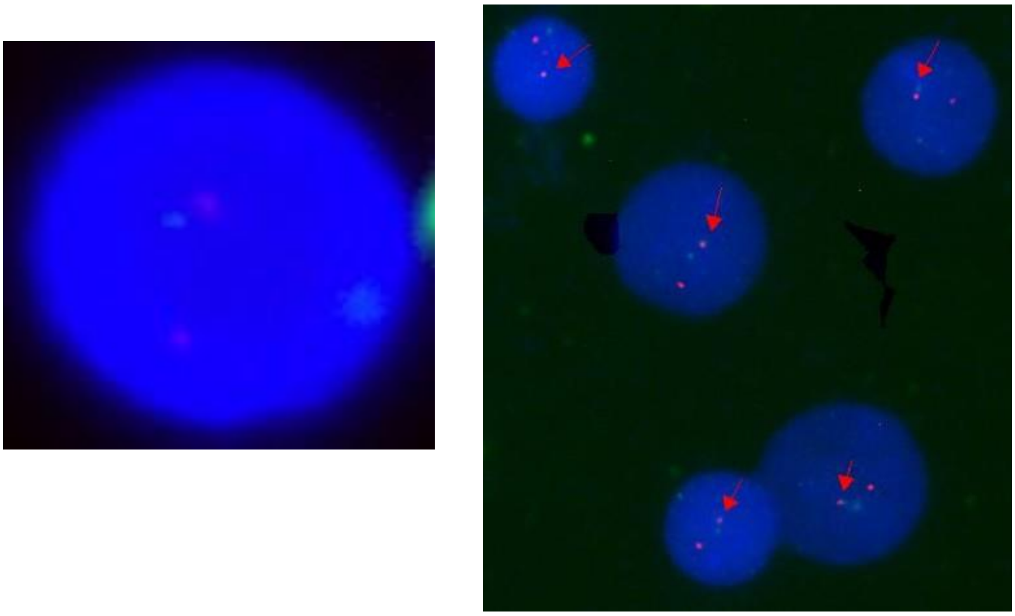

Raw figure of Figure 5L

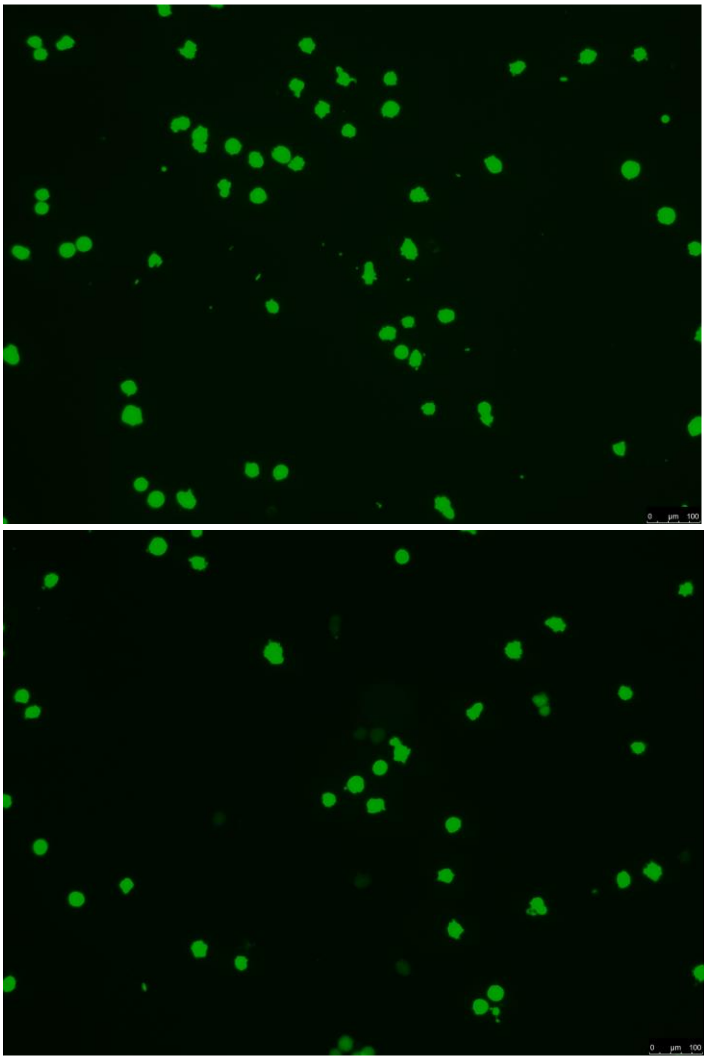

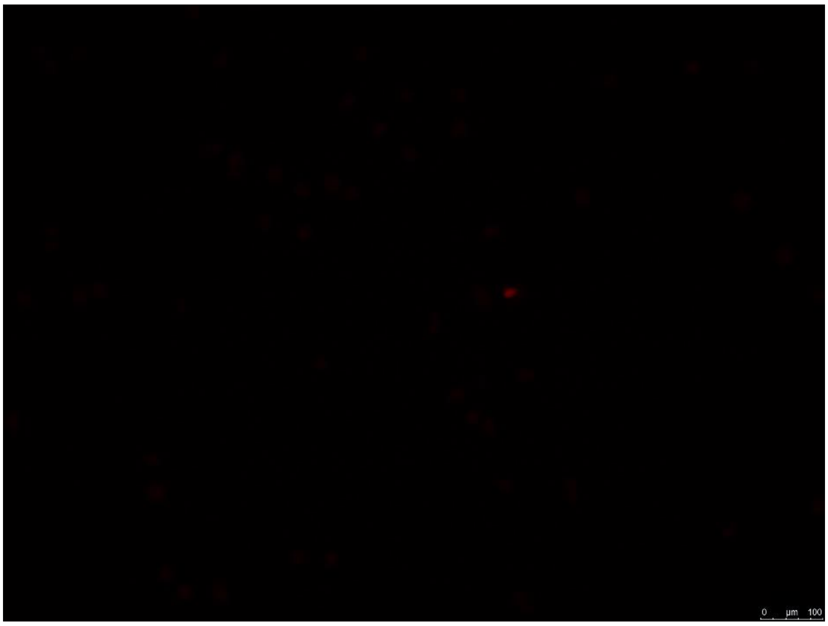
0 µm 100

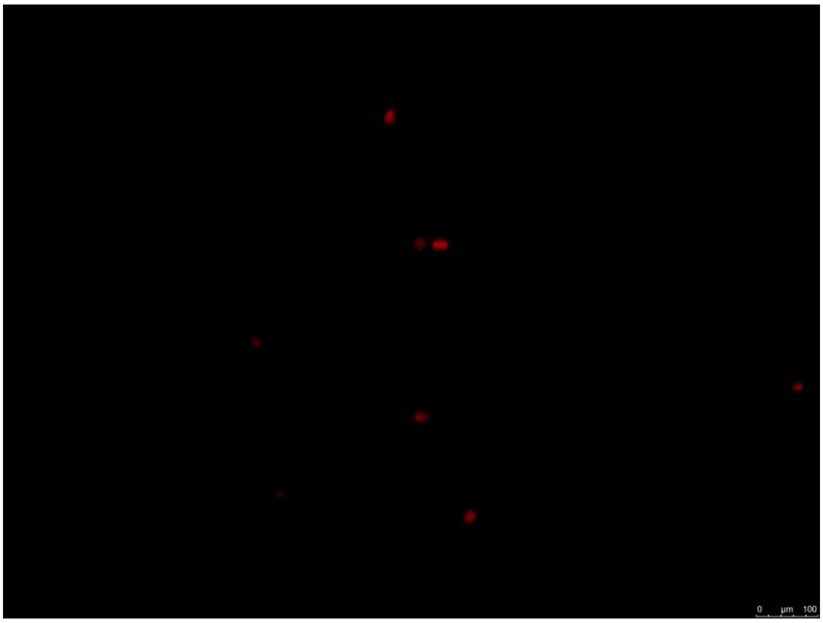
0 µm 100

Raw figure of Figure 7B- p-ERK

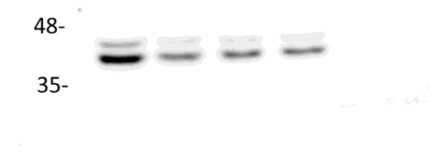

Raw figure of Figure 7B- T-ERK

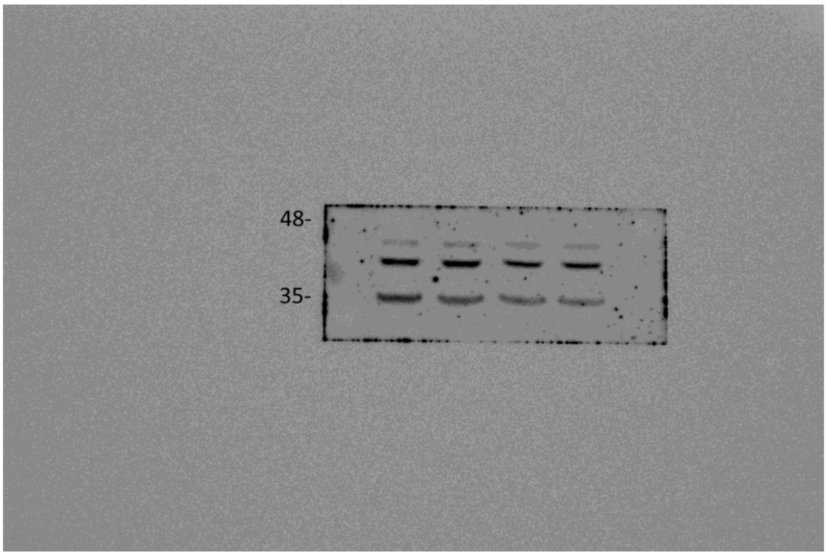

Raw figure of Figure 7B- P21

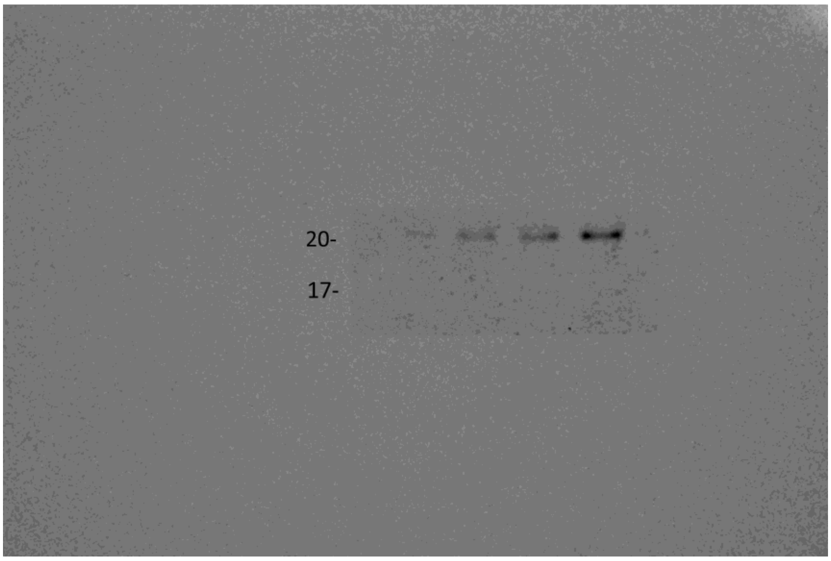

Raw figure of Figure 7B- P27

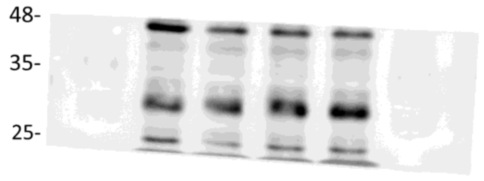

Raw figure of Figure 7B-c-PARP

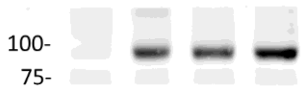

Raw figure of Figure 7B- GAPDH

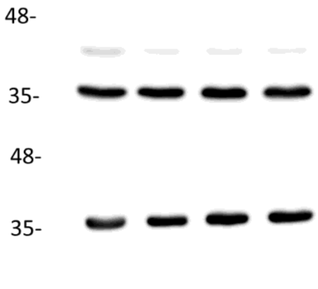

Raw figure of Supplementary figure 7C

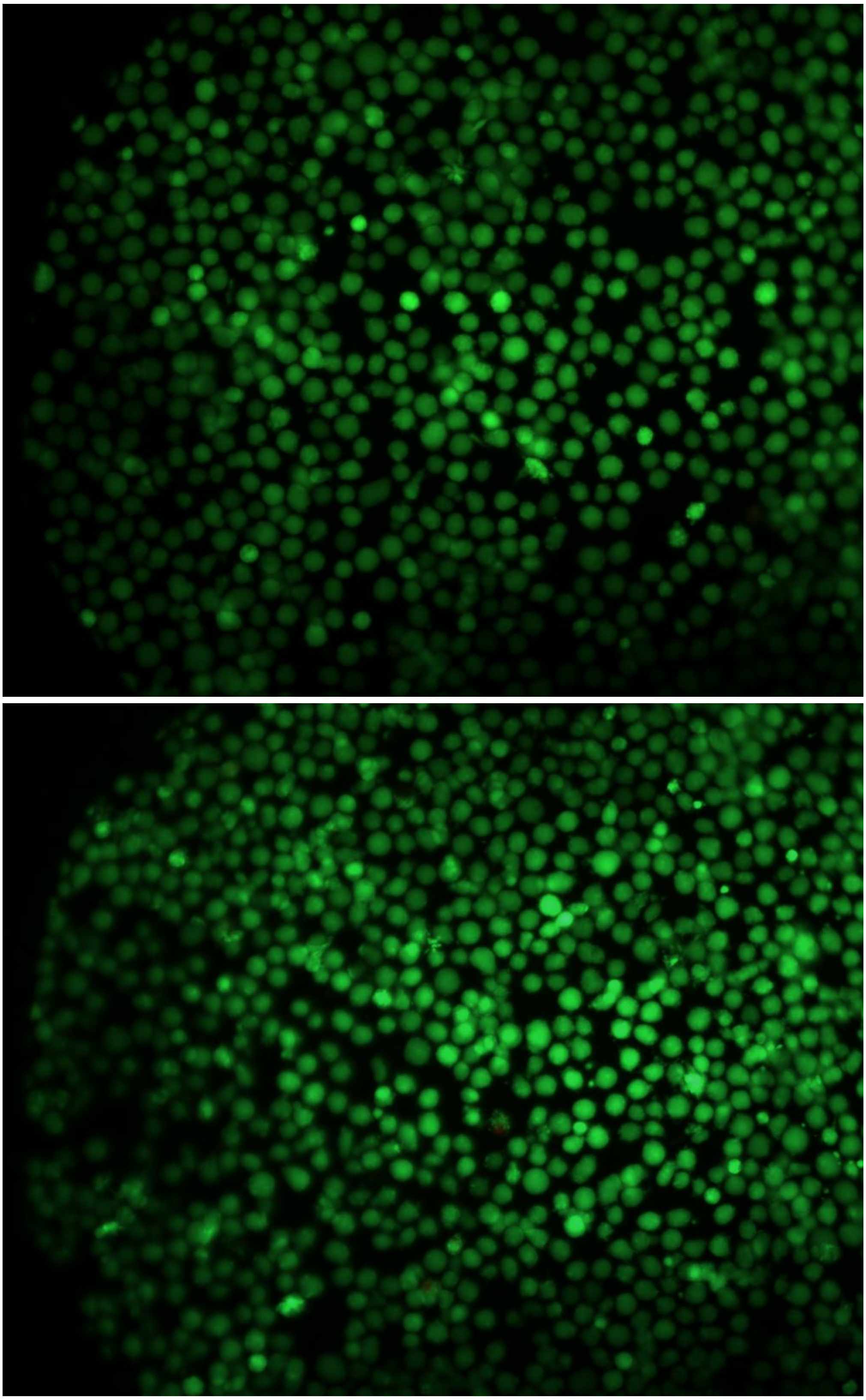

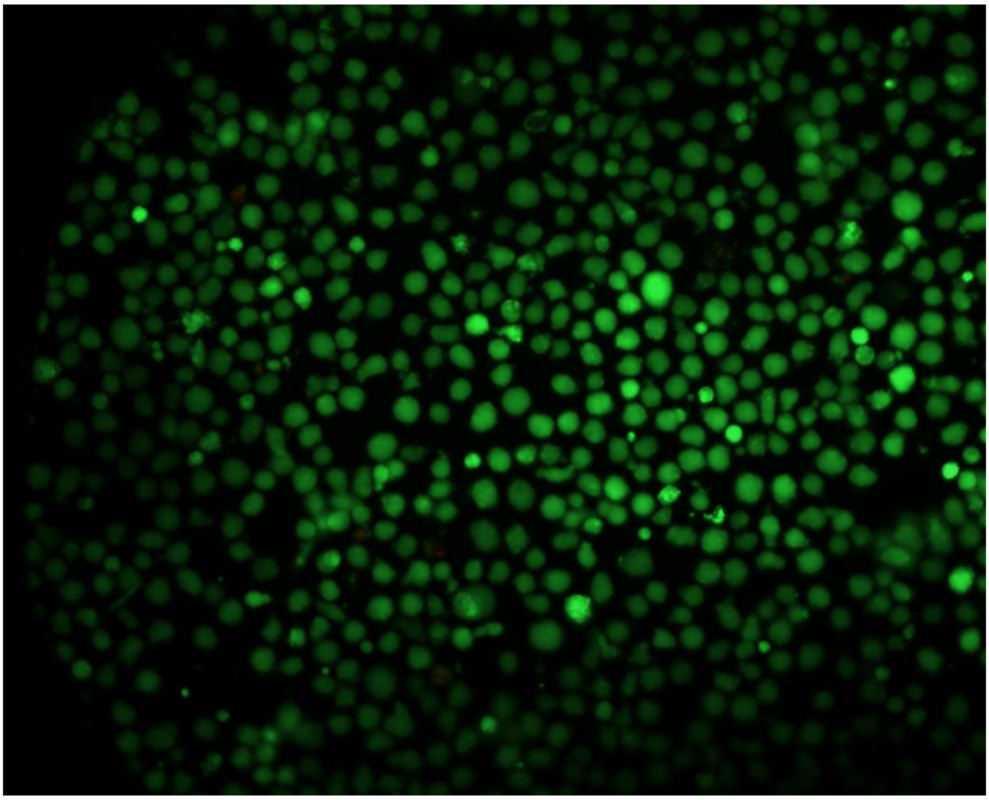

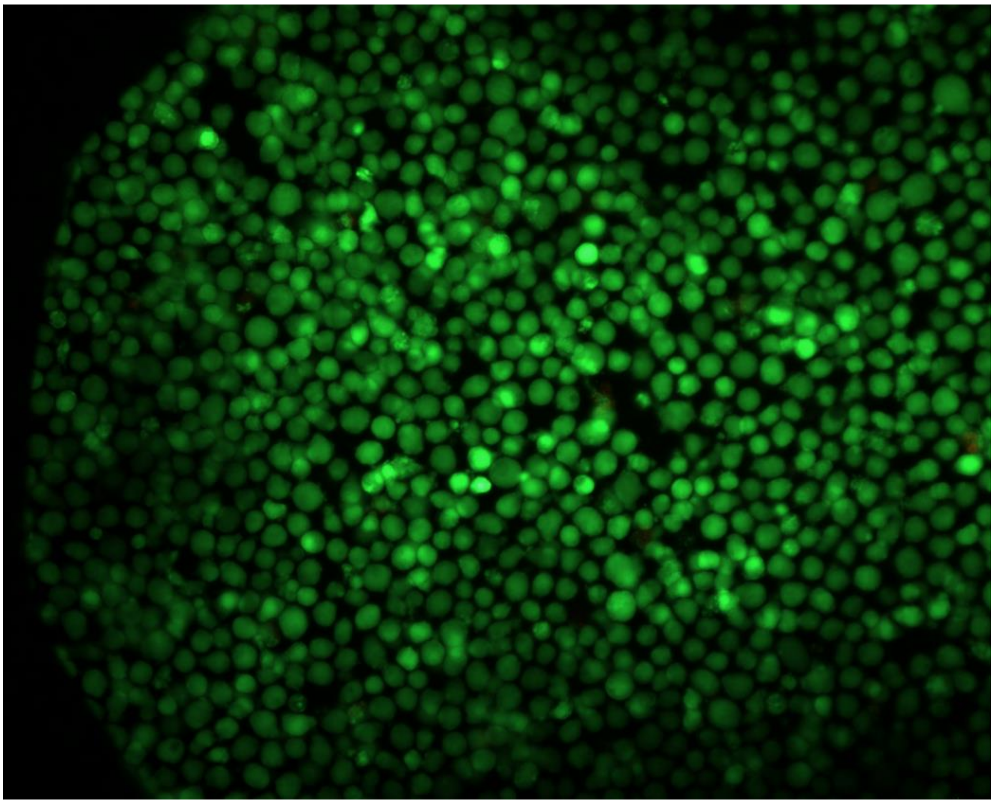

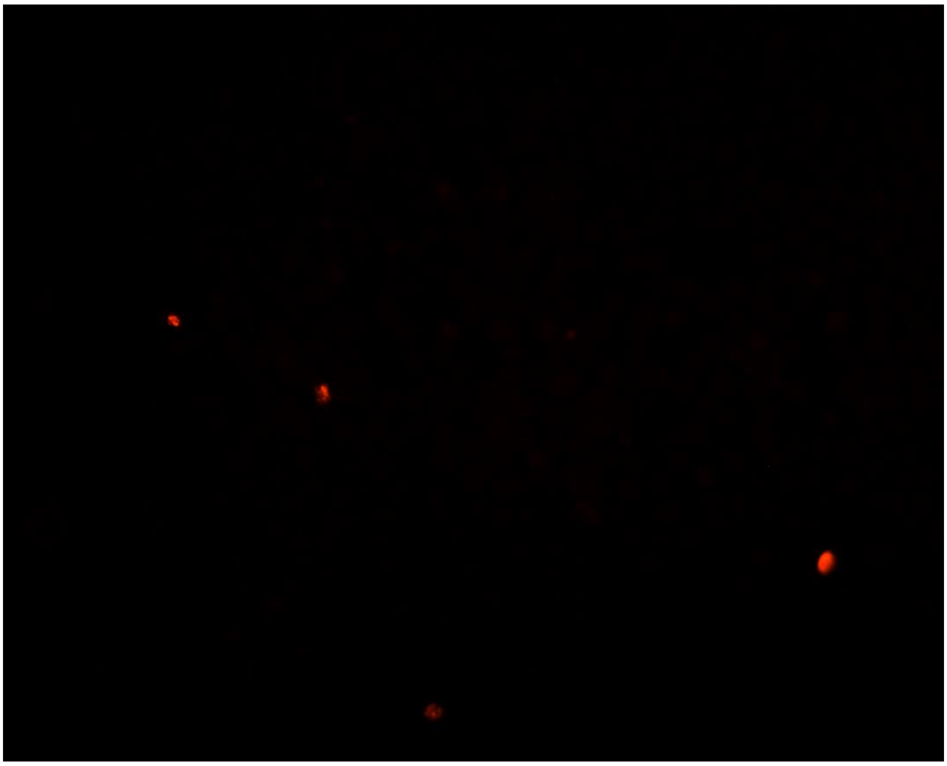

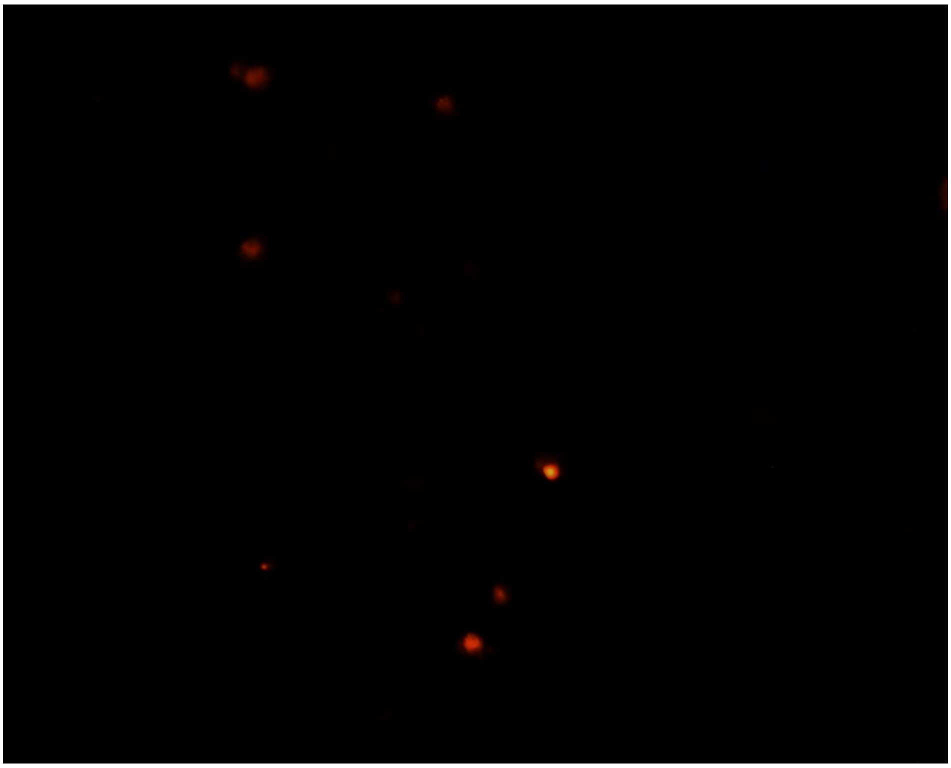

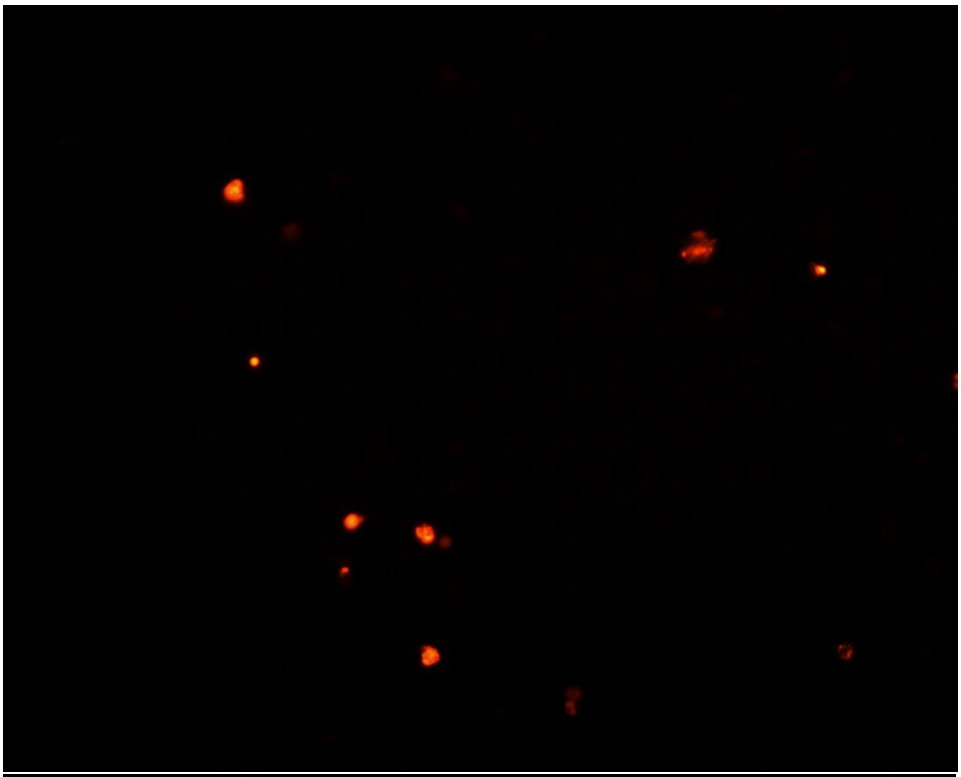

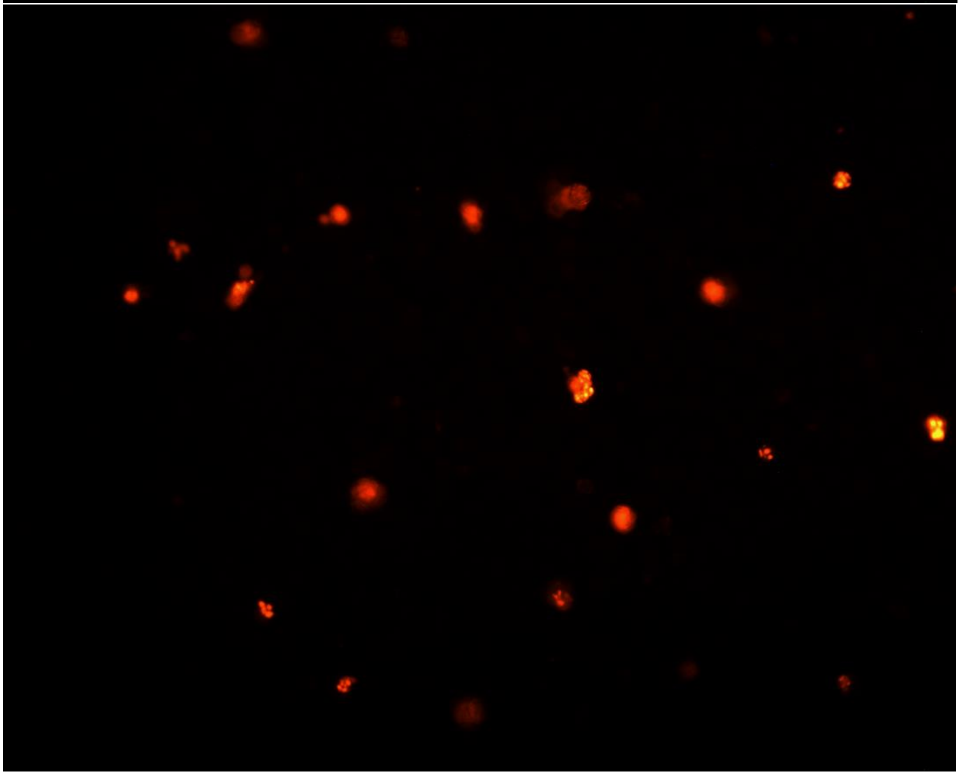

Raw figure of Supplementary figure 4

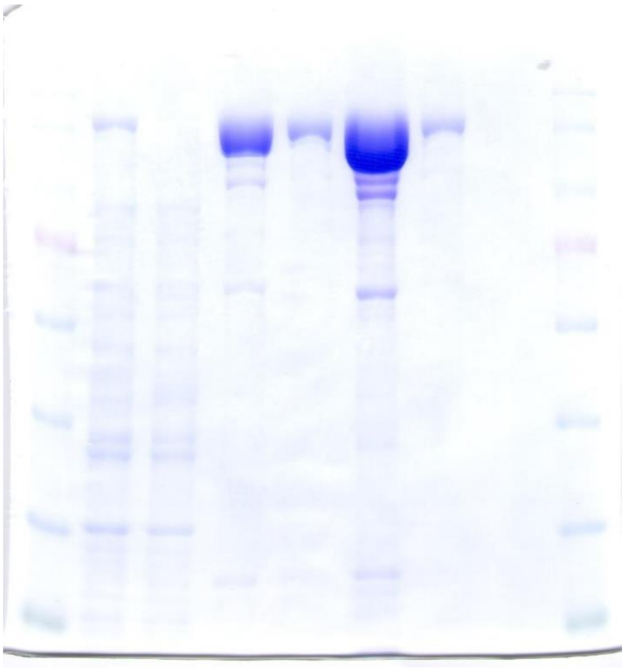

Supplement: Supplementary file 1 [file cancers-12-01399-s001.zip › File S2-Supplementary Information for whole blot.pdf]
