# Supplementary material for: ABL Genomic Editing Sufficiently Abolishes Oncogenesis of Human Chronic Myeloid Leukemia Cells In Vitro and In Vivo
Source: Cancers (Basel). 2020 May 29;12(6):1399. doi: 10.3390/cancers12061399 (PMC7352505; doi:10.3390/cancers12061399)
Supplement: Supplementary file 1 [file cancers-12-01399-s001.zip › Supplementary figures.pdf]

Supplementary Materials:

ABL Genomic Editing Sufficiently Abolishes Oncogenesis of Human Chronic Myeloid Leukemia Cells *In Vitro* and *In Vivo*

Shu-Huey Chen, Yao-Yu Hsieh, Huey-En Tzeng, Chun-Yu Lin, Kai-Wen Hsu, Yun-Shan Chiang, Su-Mei Lin, Ming-Jang Su, Wen-Shyang Hsieh and **Chia-Hwa Lee**

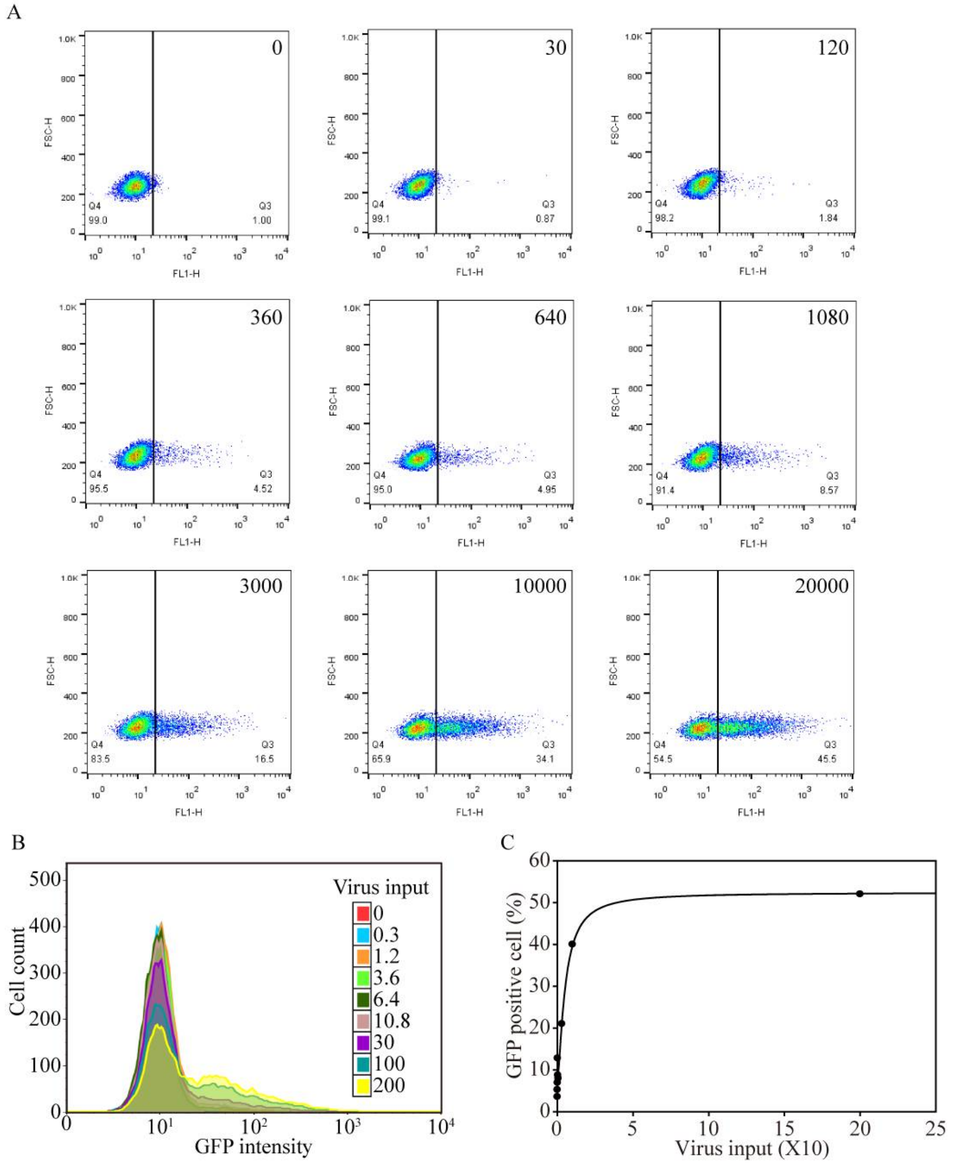

Figure 1. Optimization of viral transduction conditions for human K562 cells. GFP-positive K562 cells were analyzed by flow cytometry. Increased levels of GFP-positive cells were detected (right-shift) with increasing pLJM1-EGFP virus copy number input (fold of K562 cells) after three days. (B) The purified and concentrated PLJM1-GFP lentivirus was measured as virus copy number by qPCR analysis. K562 cells were seeded in a 6-cm dish and infected with 0.3, 1.2, 3.6, 6.4, 10.8, 30, 100 and 200:1 ratios of virus to K562 cells for three days. The GFP-positive (infected) cell population was assessed using flow cytometry. (C) Linear curve comparison of virus input and the GFP-positive cell population.

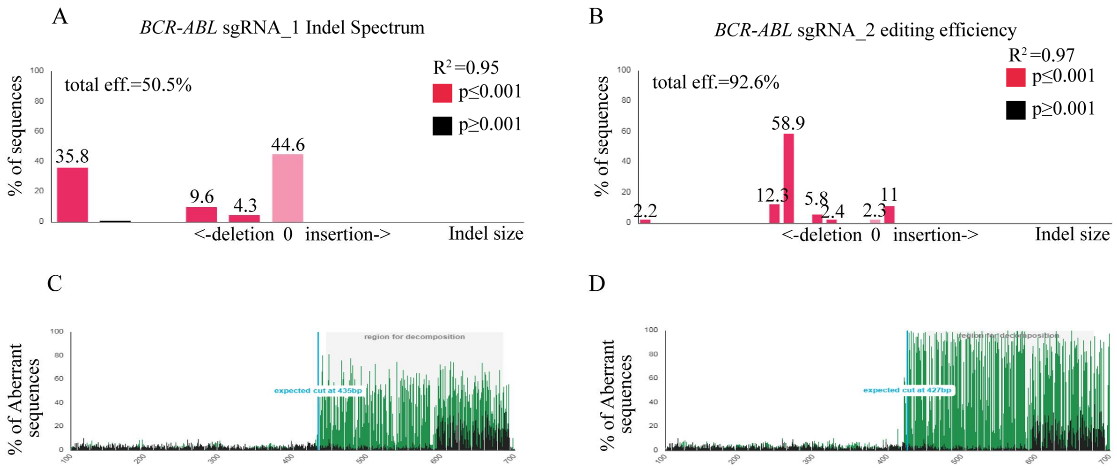

Figure 2. The TIDE algorithm analysis of *BCR-ABL* gene-targeted virus infection.The TIDE analysis of indel distribution is shown for (**A**) *BCR-ABL* sgRNA_1- and (**B**) *BCR-ABL* sgRNA_2 virus-transfected K562 cells compared to SC K562 cells. The panels illustrate the aberrant sequence signals in the SC (black), *BCR-ABL* sgRNA_1, and *BCR-ABL* sgRNA_2 (green) cell pools and the expected cleavage sites (vertical dotted line).

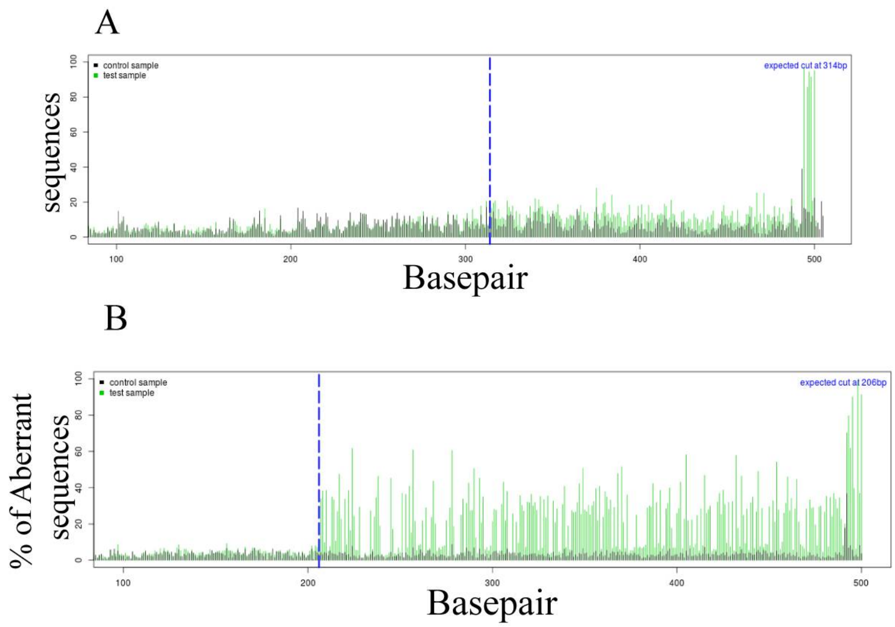

Figure S3. The original TIDE algorithm analysis of *ABL* gene-targeted virus infection. The original TIDE algorithm analysis is shown for (**A**) *ABL* sgRNA_1- and (**B**) *ABL* sgRNA_2 virus-transfected K562 cells compared to SC K562 cells. The panels illustrate the aberrant sequence signals in the SC (black), *ABL* sgRNA_1, and *ABL* sgRNA_2 (green) cell pools and the expected cleavage sites (vertical dotted line).

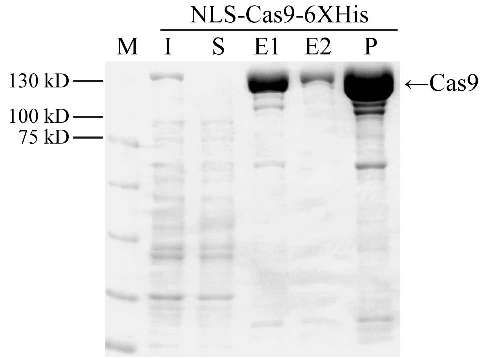

Figure S4. Cas9 protein production and purification. Cas9 protein production and purification using Ni-NTA beads and Coomassie blue staining. I: IPTG induction, S: *E. coli* supernatant, E1: Elution 1, E2: Elution 2, P: Column-purified Cas9. The predicted molecular weight of Cas9 is 140 kDa.

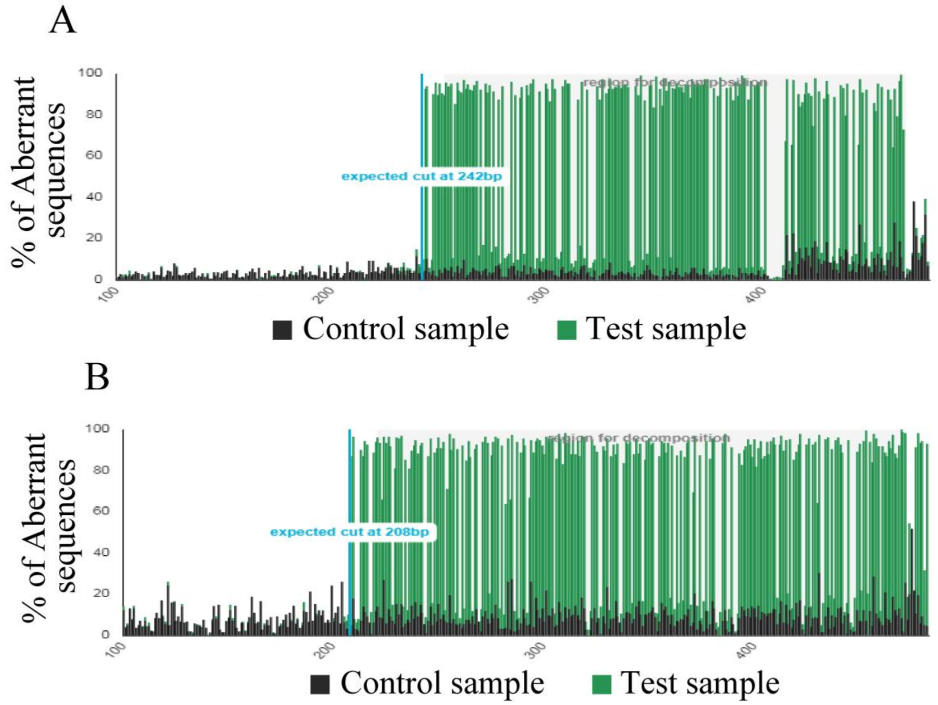

Figure S5. The TIDE algorithm analysis of *mABL* gene-targeted virus infection. The TIDE analysis of indel distribution is shown for (**A**) *mABL* sgRNA_1- and (**B**) *mABL* sgRNA_2 virus-transfected NIH-3T3 cells compared to SC NIH-3T3 cells. The panels illustrate the aberrant sequence signals in the SC (black), *mABL* sgRNA_1, and *mABL* sgRNA_2 (green) cell pools and the expected cleavage sites (vertical dotted line).

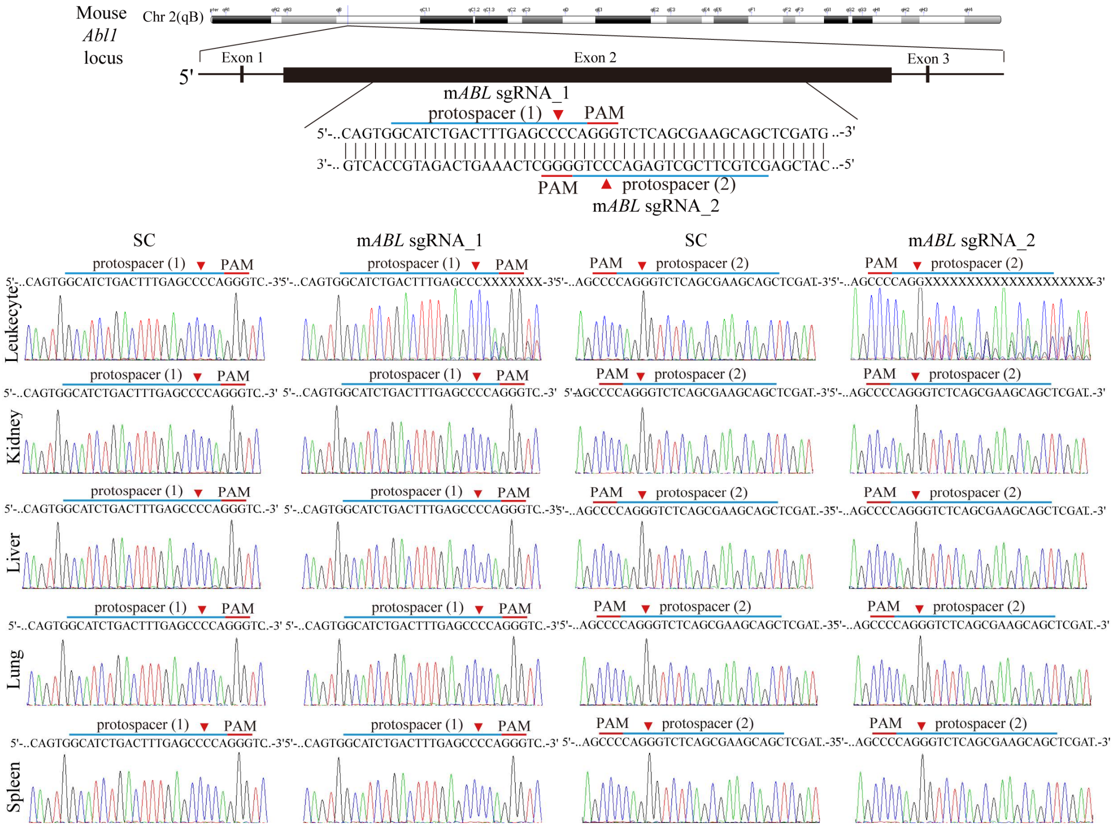

Figure S6. The representing mouse *ABL* disruptions of leukocyte and internal organs from SC- and *mABL* sgRNAs virus introduced mice. The isolated leukocytes and internal organs, such as kidney, liver lung and spleen from SC, *mABL* sgRNA_1- and *mABL* sgRNA_2 virus tail-vein injected mice were genomic analyzed for mouse *ABL* gene disruption.

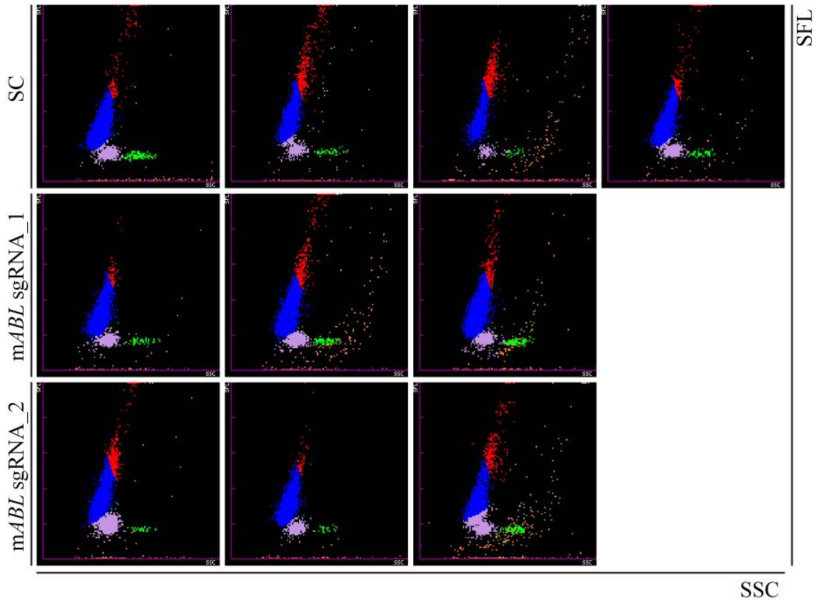

Figure S7. Hematology analysis of SC- and *mABL* sgRNAs virus injected mice. The DIFF scattergram analysis of SC ($n = 4$), *mABL* sgRNA_1- ($n = 3$) and *mABL* sgRNA_2 ($n = 3$) virus tail-vein injected mice. The leukocyte subpopulation was determined by the SSC (Side Scatter) and SFL (side fluorescence)

using IDEXX Procyte Dx hematology analyzer. The monocyte, lymphocyte, neutrophil and eosinophil cells are presented in red, blue, purple and green colors, respectively.

© 2020 by the authors. Licensee MDPI, Basel, Switzerland. This article is an open access article distributed under the terms and conditions of the Creative Commons Attribution (CC BY) license (http://creativecommons.org/licenses/by/4.0/).
